# Assessment of the Emission of Pollutants from Public Transport Based on the Example of Diesel Buses and Trolleybuses in Gdynia and Sopot

**DOI:** 10.3390/ijerph18168379

**Published:** 2021-08-08

**Authors:** Marcin Połom, Paweł Wiśniewski

**Affiliations:** 1 Division of Regional Development, Institute of Geography, University of Gdańsk, 80-309 Gdańsk, Poland; 2 Division of Landscape Research and Environmental Management, Institute of Geography, University of Gdańsk, 80-309 Gdańsk, Poland; pawel.wisniewski@ug.edu.pl

**Keywords:** trolleybus, electric bus, diesel bus, public transport, greenhouse gas emission, CO_2_ emission

## Abstract

The present study attempts to examine the research gap in terms of comparing the environmental impact of trolleybuses and diesel buses in the conditions of a country with an unfavourable energy mix. The analysed example concerns the trolleybus transport system in Gdynia, in northern Poland, which also partially serves the neighbouring city of Sopot. In the last few years, two bus lines have been electrified with trolleybuses in the In-Motion-Charging technology, which enables operation on sections without an overhead network. Using the actual operational data, a comparative analysis of the emissivity of diesel buses and trolleybuses used on the same lines in an identical operating regime was conducted. Moreover, an attempt was made to estimate the damage costs of the emission of air pollutants for the above-mentioned means of transport. Research has shown that trolleybuses significantly help to reduce emissions of nitrogen oxides, non-methane volatile organic compounds and particulate matter, while increasing sulphur dioxide emissions on the served lines. They also generate lower specific emissions of carbon dioxide compared to diesel buses. However, taking into account the differences in the number of seats in these vehicles, the length of routes resulting from a need to provide access to the necessary infrastructure and the total amount of kilometres covered on a given route, they may cause higher emissions per year and per the product life cycle than diesel buses. This is related to the unfavourable structure of energy production in Poland, which is dominated by coal sources. The research results clearly show that the use of trolleybuses in public transport contributes to a reduction of the damage costs of the emission of pollutants that amount to approximately EUR (€) 30,000–60,000 per year for the analysed lines.

## 1. Introduction

The deteriorating condition of the natural environment and climate changes related to the emission of greenhouse gases (GHG) cause concerns about the quality of further life on Earth. Therefore, measures are taken on all continents to reduce the emission of pollutants, in particular greenhouse gases [1,2]. In the last decade, international organisations have made efforts and raised the need to transform economies towards low-emission performance and energy efficiency on the global forum. Transport is one of the sectors of the economy that emit the most harmful substances. It is responsible for 25% of global carbon dioxide (CO_2_) emissions. In the 30 years since 1990, they have increased by over 70% [3]. Oil combustion is the dominant method of power supply in transport. The share of this fuel is over 90% [4]. In Poland, transport is responsible for 16.9% of the total GHG emissions. Taking CO_2_ into account, the share is even higher at 20.4% [5]. Transport in Poland is the largest source of nitrogen oxide (NO_x_) emissions (its share is currently 41% and is systematically growing). Transport is also responsible for 12% of the national emissions of non-methane volatile organic compounds (NHMC/NMVOC), 11% of particulate matter (PM) 2.5, 8% of PM 10 and 0.18% of sulphur dioxide (SO_2_) [6].

In order to reduce the emission of harmful substances to the environment, the United Nations has taken actions calling on the economies of developed and developing countries to take decisive steps in this respect [7]. The same issue, the decarbonisation of transport, has been the focus of the Organisation for Security and Cooperation in Europe [8] and the European Union (EU) that introduced a directive on the development of alternative fuels infrastructure and legal solutions for the so-called Green Deal [9,10,11,12]. The European Union funds are also a tool in the development of low-emission transport.

The European Union member states, including Poland, have been obliged to adapt their transport policies to the community’s guidelines [13]. These also concern urban public transport, which should soon become emission-free, despite the fact that Poland is a country in which the energy mix has a negative balance. Fossil fuels, in particular lignite and hard coal, account for the majority of the Polish energy market. However, the absence of emissions from public transport is critical at the point of use in densely populated areas where the quality of life matters [14]. Poland has taken up the challenges related to the transition to a resource-efficient, low-carbon economy. With this in view, it implements its own legal solutions concerning, among others, the transport sector [15,16,17,18]. The Act on electromobility and alternative fuels and The National Framework for the Development of Alternative Fuels Infrastructure exemplify legal acts in this respect [19,20]. Urban transport, which may become a model for other spheres of the society’s life and activity by switching to electric power despite the unfavourable method of electricity production in Poland [21], plays an important role in these activities.

### 1.1. Scientific Framework

Based on the presented outlines of the world and European policies regarding the development of zero-emission public transport, literature sources have been analysed in order to identify the research gap concerning a comparison of the environmental impact of diesel buses and of trolleybuses operating in the same regime. Perhaps, in the conditions of an unfavourable energy balance, the development of trolleybuses is not fully justified, and in Poland, it is less harmful to use diesel buses with a high exhaust emission standard, as electricity in Poland is produced mainly from fossil fuels [22]. So far, no comparative studies of the validity of the development of trolleybus transport have been conducted. In the world literature, one can find studies on the impact of various means of transport on the environment and on their greenhouse gas emissions [23,24]. Some of them concern public transport. Of particular significance are works by L. Klucininkas and J. Matulevicius [25] analysing GHG emissions by buses and trolleybuses in Lithuania and by L. Klucininkas, J. Matulevicius and D. Martuzevicius [26] on the life cycle costs of various public transport vehicles. The study by J. Ally and T. Pryor assesses the life cycle costs of diesel buses powered by gas and hydrogen [27]. The work by A. Lajunen [28], showing the differences in the life cycle costs of electric buses charged in different ways, is also important for the research procedure. M. Potkány et al. [29] compare the operating costs of combustion and electric buses. A. Sheth and D. Sarkar conducted similar research in India [30]. E. M. Szumska et al. [31] analysed the life cycle cost of different types of buses and alternative fuels. Many works mainly concern one means of transport or a selected technology for a given type of vehicle. The first group includes numerous papers on electric buses [32,33,34,35,36,37,38,39,40] and trolleybuses [41,42,43,44,45]. The second group includes general papers on auxiliary power technology, in particular batteries in electric buses and trolleybuses, as well as charging technologies for both types of vehicles [46,47,48,49]. Particularly noteworthy is the In-Motion-Charging technology, which builds an advantage of trolleybuses over other means of transport [50,51,52,53,54]. Additionally important are papers indicating the importance of low- and zero-emission means of transport for environmental protection [55,56].

### 1.2. Defining Research Goals

In the past, trolleybus transport was characterised by periods of dynamic development and equally rapid shutdown [57]. This was mainly affected by economic and political factors. In periods of high supply and low fuel prices, electric transport declined in importance. When problems arose on the fuel market, electric vehicles immediately grew in importance. Today, there are less than 300 trolleybus transport systems worldwide [58]. However, trolleybus transport is gaining in importance again, and it is an addressee of political activities. New systems are being built in Pescara and Prague and designed in Berlin and Iasi [21]. However, the key questions remain regarding the actual environmental impact of individual public transport modes and their possible impact on adverse climate change. This issue is particularly important in countries such as Poland where energy electricity is mainly produced from fossil fuels. The question then arises whether trolleybuses are actually the best solution for public transport in cities. The main research hypothesis assumes that even in the conditions of an unfavourable energy mix, in the life cycle of a vehicle, trolleybuses are a better solution than buses powered by diesel oil. In order to achieve the assumed research goals, additional questions were asked:

1. Is trolleybus transport powered by electricity from lignite and hard coal still environmentally friendly?
2. Are trolleybuses a better alternative than diesel buses in Polish conditions?
3. What is the environmental impact balance of diesel buses and trolleybuses in the same regime of operating on the same lines?

## 2. Materials and Methods

**Figure 1:**
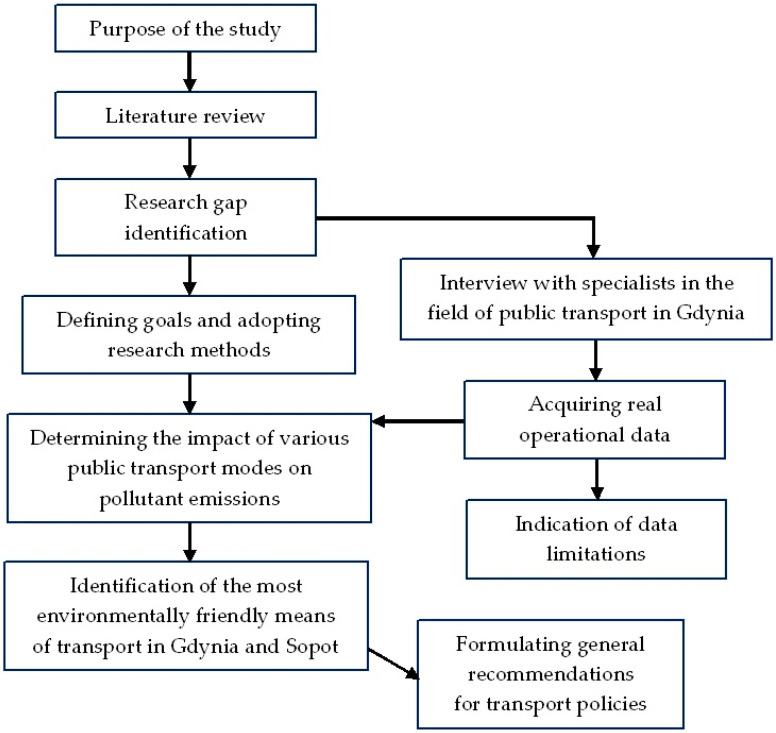
Scheme of the research procedure (source: Own elaboration).

Along with the advancing climate changes, trolleybus transport is again becoming a subject of research. Due to the low popularity of this means of public transport, there are not many scientific studies that deal with the impact of using trolleybus on the natural environment. Comparative studies of various means of transport take a special place in this respect. Valuable studies that show the real impact of urban transport on the environment and on the emission of pollutants from particular types of vehicles are relatively few. The development of Gdynia’s trolleybus transport through theelectrification of bus lines on which diesel-powered buses have run or still run gives a unique opportunity to make calculations based on the actual operational measurements. The study was designed based on the procedure presented in Figure 1.

The influence of various means of transport operating in the same traffic conditions enables formulating universal recommendations for the development of public transport. After defining the main goal, in-depth literature studies were conducted using the desk research method. As a result, the research gap in the comparison of emissions of pollutants by trolleybuses and diesel buses in real operating conditions was confirmed. On this basis, the goals were defined, and the research methodology was adopted. Simultaneously, actions were taken to obtain real operational results from the transport organiser (the Public Transport Authority in Gdynia) and transport operators (for trolleybuses, from the Trolleybus Transport Company in Gdynia, and for buses, from he Municipal Transport Company in Gdynia and private carriers). Based on the obtained operational data, calculations were made of the actual impact of trolleybuses and diesel buses operating on the same lines throughout one year and for the vehicle life cycle. Indicating limitations in operational data that were used to compare both means of transport is an additional effect of the study. As a result of the conducted research procedure, the real best way to develop urban transport in Gdynia and Sopot conditions was identified, and conclusions for transport policies were formulated.

The emission of pollutants on selected lines for diesel buses and trolleybuses was estimated with the use of the pollutant emission and climate cost calculator for public transport, which is a tool for calculating pollutant emissions recommended by the Centre for EU Transport Projects [59]. It is a unified tool for all transport projects in Poland, previously used by [24,60] to evaluate the environmental and economic effects of electromobility in sustainable urban public transport. The pollutant emission and climate cost calculator shows how to calculate emissions for buses and other public transport based on fuel and energy consumption. This calculation should be used when different powered modes of transport are compared with each other (e.g., ON, CNG and electric buses). The data on exhaust emissions for individual EURO standards are the maximum emissions for a given standard in accordance with the indicated legal acts.

To analyse the environmental effects, the amount of carbon dioxide (CO_2_), nitrogen oxides (NO_x_), non-methane volatile organic compounds (NHMC/NMVOC) and particulate matter (PM) were calculated. In the case of diesel buses, these are emissions generated at the place of use. In the case of trolleybuses, these are emissions generated outside the place of use, in course of the production of electricity necessary to power them. For lines served by trolleybuses, emissions of sulphur dioxide (SO_2_), also produced during energy production, were calculated. For the most up-to-date results, the calculations used the updated emission factors for CO_2_, SO_2_, NO_x_ and total dust for electricity, based on the information contained in the national database on greenhouse gas emissions and other substances for 2019 [61]. These are as follows: 758 kg/MWh for CO_2_, 0.539 kg/MWh for SO_2_, 0.608 kg/MWh for NO_x_ and 0.031 kg/MWh for total dust. For NHMC/NMVOC, the emissivity ratio was assumed at the level of 1.4 g/GJ (0.00504 kg/MWh), defined for Poland according to the Ricardo-AEA report [62]. The average emissivity ratios generated by diesel buses were obtained based on the guidelines of EU directives and regulations, depending on the EURO standard that the rolling stock meets (Table 1).

Based on the calculated emissions of pollutants, the external costs of emissions for selected diesel buses and trolleybus lines were estimated in accordance with the IPA (Impact Pathway Approach) method recommended by the European Commission. It is the most modern way of assessing the effects of air pollution, taking into account the costs of damage and willingness to pay. This method tracks emissions of pollutants and identifies their impacts, then assesses the impact of the emissions on human health, the environment and economic activity and quantifies the damage caused (in monetary amounts) [63]. For this purpose, the indicators specified for Poland, as defined in the updated Handbook on the external costs of transport (Version 2019–1.1) [64], were used with the following formula:

(1) $$PCV=\underset{i}{\sum }\underset{j}{\sum }PC_{ij}\times Q_{ij}$$

where:

*PVC*—cost of air pollution [€/kg],
*PC_ij_*—cost of pollution [€/kg],
*Q_ij_*—amount of pollution [t],
*_i_*—pollution type,
*_j_*—line type.

When estimating the damage costs of air pollutant emissions on the tested lines, harmful substances affecting the quality of life of the area residents were taken into account. The quality of life of the society largely depends on the concentration of air pollutants in the area of their residence. Therefore, in accordance with the developed methodology for estimating the external costs of air pollution emitted from road transport at a national level, it was limited to local pollutants. Thus, the SO_2_, NHMC/NMVOC, NO_x_ and PM pollutions that cause local health effects, material and construction damage and loss of biodiversity were taken into account. 

Taking into account the fact that estimation of the emission of air pollutants and the related external costs is also affected by the parameters of the lines selected for the study (e.g., the route length determined by access to the necessary infrastructure), by the number of trips (related to with the number of seats in vehicles) and by the sum of kilometres covered, in the last stage of the study, simulations of the emissivity and costs of emission damage were carried out in several variants, assuming that trolleybuses are used on the lines currently served by diesel buses, and diesel buses are used on the lines currently served by trolleybuses. The obtained results were compared with the emission values and costs generated in real conditions, with the current operation. In order to eliminate the impact of various parameters of individual routes indicated in Section 4.2, as well as of the differences in the number of trips and kilometres covered by the analysed vehicles, simulations of the emission of air pollutants and the related damage costs were conducted in several variants. It was assumed that in variant 1 (V_1_), line 32 is served by diesel buses instead of trolleybuses; in variant 2 (V_2_), line 170 is served by trolleybuses instead of buses; in variant 3 (V_3_), line 181 is served only by buses; in variant 4 (V_4_), line 181 is served only by trolleybuses. The obtained results were compared with the emission levels and costs generated on these lines in real conditions of the current operation (Table 2 and Table 3).

## 3. Description of the Case Study

### 3.1. Gdynia’s Experience in the Development of Public Transport Based on Low-Emission and Zero-Emission Vehicles

Urban transport in Gdynia, in northern Poland, which also partially serves neighbouring municipalities, including Sopot, is set as an example of proper management and development based on paradigms of limiting the environmental impact and on friendliness to passengers. For almost two decades, the municipality of Gdynia, as the owner of urban public transport, has been actively using external funds for its modernisation and development. Trolleybus transport, as particularly underinvested and threatened with closing down still at the end of the 20th century, has undergone a long transformation towards a system that is presented as a model in Europe [65,66,67,68]. Actions were taken in Gdynia to introduce low-floor trolleybuses converted from second-hand buses [69]. It was also one of the first systems in Europe to base its development on trolleybuses equipped with on-board batteries [70]. Nowadays, Gdynia and Sopot are served by 16 regular public transport lines and 3 seasonal lines [71]. On eight regular lines and one seasonal line, there are sections of routes without overhead lines, where trolleybuses run with power from on-board batteries [72]. Thanks to good experience in the operation of trolleybuses in the In-Motion-Charging technology, further electrification of bus lines is planned [73].

In 2007, the authorities of Gdynia decided to base city transport on two environmentally friendly solutions. In addition to the existing trolleybus system, they decided to put CNG (compressed natural gas) powered buses into operation. Because of the division of municipal carriers in Gdynia into one trolleybus and two bus ones, gas-powered vehicles were delivered to the Municipal Transport Company (PKM) [74]. The second bus operator in Gdynia (Bus Transport Company–PKA) used only diesel buses and mainly served the northern districts of the city, where there are also no trolleybus connections. Due to the disproportions in access to low-emission and zero-emission transport of residents of northern districts of the city, the latest implementation plan for the operation of electric buses was directed to the Bus Transport Company (PKA) in the northern part of the city. Previously, the operator of electric vehicles was the Trolleybus Transport Company (PKT) [75]. Further use of this carrier’s experience could facilitate putting electric buses into operation.

### 3.2. Transforming a Bus Line into a Trolleybus Line in Gdynia and Sopot

**Figure 2:**
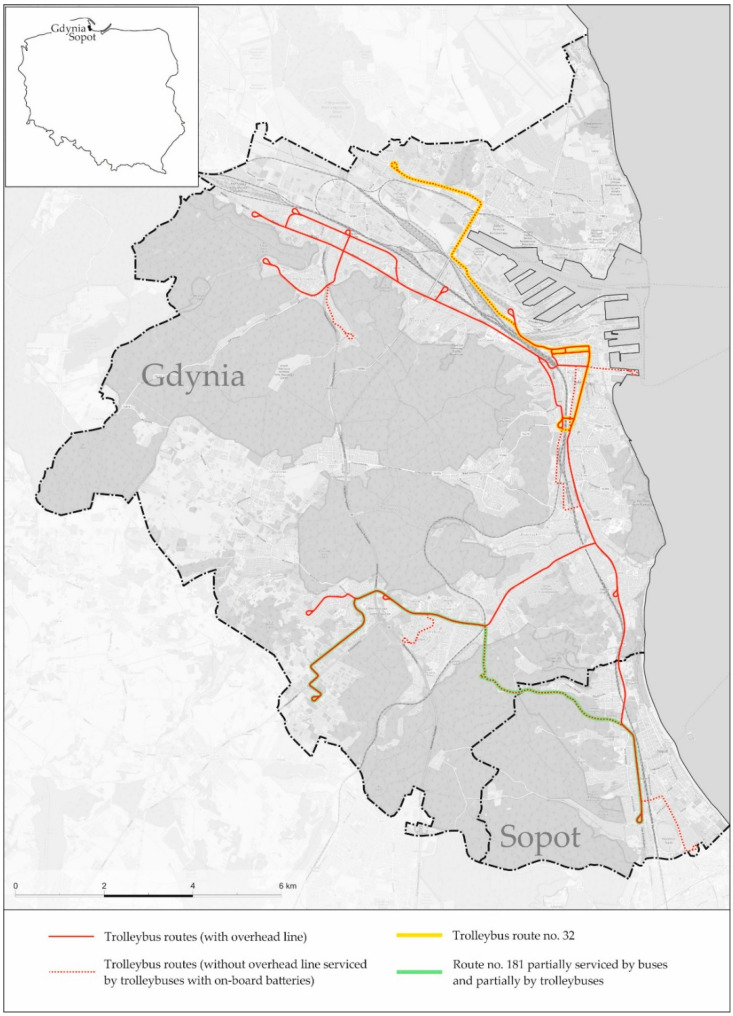
Connection diagram for public transport lines 32 and 181 in Gdynia and Sopot (source: Own elaboration based on OpenStreetMap).

Gdynia’s public transport system has been developed in recent years based on environmentally friendly means, such as CNG buses and trolleybuses. The electrification of bus lines with trolleybuses was of particular importance in this process. Thanks to the use of on-board batteries as an alternative source of powering trolleybuses and charging them, the In-Motion-Charging technology helped to implement electric transport on several bus lines [54]. Two examples are crucial to illustrate the policy of public transport development in Gdynia and Sopot. Bus line 170 (next transformed into a trolleybus line 32), whose route ran from Kaszubski Square in the city centre to the Pogórze Dolne district located in the northern part of Gdynia, became fully electrified (Figure 2). Thus far, this part of the city had been deprived of access to electric transport and, to a large extent, also to low-emission buses powered by CNG [71]. Trolleybus connections could not be launched because there was no trolleybus traction infrastructure, which was removed from this part of the city in 1972 [76].

**Figure 3:**
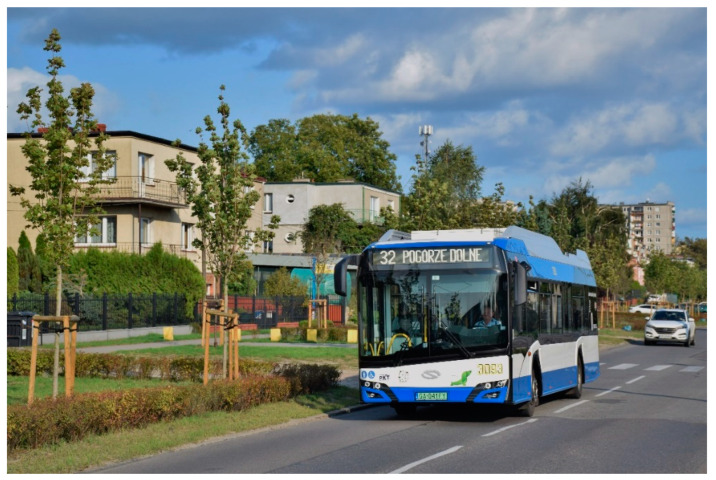
A trolleybus running on line 32 using battery power in Gdynia (photo taken by M. Połom).

The gas-powered buses are operated by a municipal carrier, which has its depot in the southern part of the city and mainly serves routes in the southern and central districts. Therefore, there was a major disproportion in access to low- and zero-emission vehicles in Gdynia. This problem could be solved along with the rising popularity of auxiliary power sources in trolleybuses, thanks to which it is possible to extend the route with sections devoid of an overhead line [77]. Due to the distance of the northern districts of the city from the trolleybus overhead network, the introduction of trolleybuses to service bus lines became possible only with the development of the In-Motion-Charging technology [51,53,78]. This technology was used to electrify bus line 170, for which six new trolleybuses equipped with lithium-ion batteries with a capacity of 87 kWh were earmarked [79]. In order to facilitate connecting and disconnecting vehicles from the overhead contact line, an additional branch with a length of 200 m was built, and the route in the city centre was extended so that the trolleybuses could recharge the on-board batteries (Figure 3). The line is now marked as number 32 and has been electrified since September 2020. 

**Figure 4:**
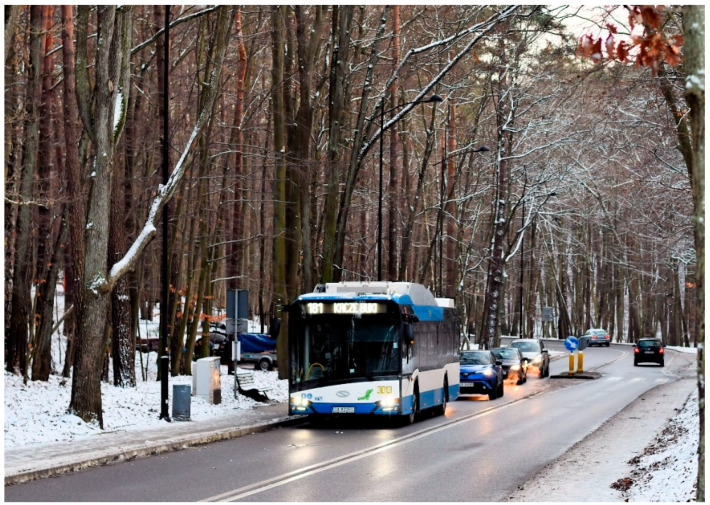
A trolleybus running on bus line 181 using battery power in Sopot (photo taken by K. Grzonka).

The second example is the partial electrification of bus line number 181 connecting the southern districts of Gdynia with Sopot (Figure 4). As in the case of line 170, it is a route with a typical frequency every 15 min. However, unlike route 170, which is operated by traditional 12-m vehicles, on weekdays, line 181 is serviced with articulated buses. 

Most of the vehicles were powered with diesel oil and were compliant with the Euro 4 and Euro 5 emissions standard. CNG-powered buses also covered some of the routes. Due to the course of a significant part of the route under a trolleybus overhead line, both in Gdynia and Sopot, this line was selected for possible electrification with the use of trolleybuses equipped with on-board energy storage [74]. Due to the fact that part of the route runs through the Tricity Landscape Park, it was particularly important that environmentally friendly vehicles operate on this line, but the construction of a classic trolleybus infrastructure was impossible due to the lack of roadsides, trees growing along the road edge and numerous curves.

## 4. Results and Discussion

### 4.1. Characteristic of the Functioning of the Analysed Lines

The article analyses two public transport lines in Gdynia and Sopot that have been electrified in recent years. To serve them, trolleybuses equipped with on-board batteries were implemented, which allowed reducing investment costs by not having to build a trolleybus overhead line. The two lines mentioned in the earlier chapters are line 170, which has been renamed as no. 32, and trolleybus line 181, which is jointly operated by trolleybuses and buses. The former one is fully served by trolleybuses, and in the case of the latter one, trolleybuses perform basic all-week and day-long tasks, while the so-called peak extras are supplemented with buses with diesel engines. The operational characteristics of both lines are presented in Table 4.

Line 32 is served with Solaris Trollino 12 M trolleybuses, manufactured in 2020 and equipped with LTO batteries with a capacity of 87 kWh. On line 181, there are trolleybuses equipped with lithium-ion batteries, manufactured in 2018–2019. Standard vehicles (12 m long) have batteries with a capacity of 58 kWh, and articulated ones, 87 kWh. Before the electrification of bus line 170, its operation was ensured by standard buses with a length of 12 m, manufactured in 2005–2015 and meeting the Euro 3–5 exhaust emission standard. On weekdays, line 181 is additionally served by articulated buses that meet the Euro 5 exhaust emission standard. They were produced in 2011–2016.

The average annual fuel and electricity consumption was assumed for the calculations. In the case of trolleybus line 32, due to its shorter period of operation, the average electricity consumption was calculated from September 2020 to March 2021. In the case of line 181, the average value of electricity consumption was calculated for the entire year, taking into account the different sizes of rolling stock serving the route on weekdays and at weekends.

### 4.2. Comparison of the Emissions of Pollutants and Damage Costs of the Emission of Air Pollutants from Trolleybuses and Diesel Buses on the Analysed Lines

The conducted calculations show that the unit emission of CO_2_ from trolleybuses is lower by approximately 15% for line 32/170 and by approximately 7% for line 181. Very clear differences are noticeable in the case of other pollutants, in particular NHMC/NMVOC and NO_x_. Trolleybuses are also characterised by twice-lower unit emissions of suspended dust compared to diesel buses. However, they are a source of SO_2_ emissions produced outside the place of their use (Table 5).

Taking into account total annual CO_2_ emissions, in the case of the tested lines, it is higher for trolleybuses by 63,802.16 kg in comparison to the emission from buses on line 32/170, and it is 57,448.02 kg higher than on bus line 181 (Table 6). In the case of CO_2_ emissions on the tested lines, across the entire life cycle of vehicles, the differences are even greater. The emission of carbon dioxide in the life cycle of trolleybuses in comparison to the life cycle of buses is 4037.04 Mg higher on line 32/170 and 37,873.5 Mg higher on line 181. It should be emphasised, however, that the level of operational readiness, reliability and lifetime of trolleybuses are significantly higher than those of buses [76,80]. Therefore, in the conducted calculations, the service life of buses was assumed to be 12 years, while for trolleybuses it was 20 years. However, this does not change the fact that trolleybuses on the tested lines are characterised by higher CO_2_ emission in the vehicle’s life cycle in relation to diesel buses, even when the service life is averaged. This is influenced by the differences in the length of routes covered by trolleybuses and buses after electrification of the tested lines and adjustment of timetables, the number of performed trips and the total sum of kilometres covered, as presented in Table 4. However, this situation also results from the unfavourable structure of electricity production based on coal sources. As indicated in the data of the Energy Market Agency, in January 2021, 72% of electricity in Poland was generated in hard coal and lignite-fired thermal power plants, and only 13.8% came from renewable energy sources [81]. With such a structure of energy production, the environmental burden is shifted from the stage of using the trolleybus to the stage of producing the energy carrier, as noted, among others, by Ma et al., and Chłopek and Lasocki [82,83], who compared greenhouse gas emissions in the life cycle of battery-powered electric vehicles and diesel vehicles.

As results from the conducted calculations, the use of trolleybuses in public transport contributes to a significant reduction in the emission of other air pollutants in relation to diesel buses, both on an annual scale and in the entire life cycle of vehicles. This applies in particular to NHMC/NMVOC and NO_x_ emissions (Table 6). The obtained results regarding emissions of CO_2_ and other air pollutants from public transport based on the example of trolleybuses and buses in Gdynia and Sopot are similar, among others, to the results of research on the environmental effects of electromobility in sustainable urban public transport in another Polish city—Szczecin [24]. Therefore, trolleybuses can be considered zero-emission vehicles at the point of use. However, with an unfavourable structure of electricity production necessary to drive them, and taking into account the differences in the number of seats in vehicles, the number of trips and often extended travel routes due to the need to ensure access to the necessary infrastructure, throughout their life cycle, they can have higher CO_2_ emissions than diesel buses, with significantly lower emissions of NHMC/NMVOC, NO_x_ and PM. Yet, they are also a source of SO_2_ emissions.

The emission of air pollutants can lead to different types of damages. Most relevant and probably best analysed are the health effects resulting from air pollution. However, other damages are also relevant, such as building and material damages, crop losses and biodiversity loss. Costs of air pollution are one of the external cost categories that has been analysed the most [64]. The external costs of transport are part of social costs and are not reflected in market prices, nor in the costs of all road users [63,84]. The use of trolleybuses as a means of public transport significantly reduces the costs of local damage due to air pollutants. The estimated total external costs of the emission of air pollutants from trolleybuses in relation to diesel buses on the tested lines (based on the IPA methodology described in Section 2) are nine times lower in the case of line 32/170 and over five times lower in the case of line 181 (Table 7). Taking into account the entire life cycle of vehicles, despite the differences in their service life, these differences amount to € 655,940 for lines 32/170 and € 303,360 for line 181 in favour of trolleybuses.

### 4.3. Simulations of the Emission of Pollutants and Damage Costs of the Emission of Air Pollutants on the Tested Lines in Various Variants and Their Comparison with Real Conditions

The conducted simulations showed that if line 32 was still operated by diesel buses, the annual CO_2_ emissions would be higher by over 70,000 kg, NHMC/NMVOC by over 800 kg, NO_x_ by almost 6000 kg and PM by almost 20 kg. Such a solution would only allow avoiding the emission of 290.49 kg of SO_2_. When comparing the emission of air pollutants in the life cycle of diesel buses and trolleybuses (including a lower value of carbon dioxide emission in the case of buses), one should take into account the difference in the service life of these vehicles, as described earlier. Leaving diesel buses on line 32 would generate additional damage costs of the emission of air pollutants amounting to € 85,700 per year.

In turn, the introduction of trolleybuses to operate on line 170 would allow avoiding over 50,000 kg CO_2_, almost 600 kg NHMC/NMVOC, over 4000 kg NO_x_ and almost 14 kg of particulate matter. It would also reduce the external costs of the emission of air pollutants on this line by € 61,700 per year.

The conducted simulations showed that in the case of line 181, currently operated partly by diesel buses and partly by trolleybuses, its full coverage only with diesel buses would result in an annual increase in carbon dioxide emissions by 30,600 kg, non-methane volatile organic compounds by 713.69 kg, nitrogen oxides by over 3100 kg and particulate matter by 30.81 kg, while reducing sulphur dioxide emissions by 274.69 kg. The annual costs of damage due to the emission of air pollutants would increase by almost € 39,700. Using only trolleybuses on line 181 would, in turn, reduce CO_2_ emissions by less than 23,000 kg, NHMC/NMVOC by 566 kg, NO_x_ by 2221 kg and PM by less than 614 kg, with an increase in SO_2_ emissions by 217.84 kg per year. The annual costs of damage due to air pollutants when operating this line only by trolleybuses would be €31,500 lower than today.

## 5. Discussion

Gdynia’s public transport system can be a European example of the effective implementation of pro-ecological solutions based on CNG buses and trolleybuses. Of particular importance in this process was the electrification of bus lines with trolleybuses, whose importance in serving large urban centres should increase, for example, in light of the projected reduction in transport emissions as part of the implementation of the European Green Deal.

The problem of pollutant emissions is of key importance for the assessment of the legitimacy of the functioning and development of individual public transport means. There are opinions in the public discourse that since the energy mix is unfavourable, i.e., electricity is produced from high-emission sources, e.g., hard coal and lignite, the development of electric transport is unfounded. The article attempts to verify this assumption. This is especially important for developing countries and those where fossil energy is still important. In the European Union, this issue mainly concerns the countries of Central and Eastern Europe, as well as the non-EU countries of the former Soviet Union (USSR) [21]. In these countries, electrified urban transport is still highly developed, and at the same time, electricity is largely produced from coal.

The article attempts to analyse the unique situation to compare the emissions from the operation of combustion buses and trolleybuses running on the same communication lines in Gdynia. So far, the literature on the subject primarily compares electric buses, diesel buses or trolleybuses [24,25,26,27,28,29,30,31,32]. Occasionally, the operation of different types of vehicles in one city was assessed, but these were not the cases of working in the same regime, on the same lines. In the case of Gdynia, due to the transport policy adopted by the local government authorities, bus line 170 (currently trolleybus line 32) was completely electrified and bus line 181 was electrified for the most part. Thanks to these activities, it was possible to collect comparable data.

The presented study showed the possibility of making comparisons between different means of public transport. It is also a starting point for further considerations in terms of the overall cost of life of a vehicle, including the production and disposal of vehicles and infrastructure. In the next stage of research, an attempt should be made to calculate these indicators. It is also worth conducting comparative studies of electric buses and trolleybuses. The first of these means of public transport is currently gaining an advantage resulting from political decisions. However, there is no real presentation of the environmental costs of operating electric buses in the conditions of the Polish electricity market.

## 6. Conclusions and Recommendations

The conducted research showed that replacing diesel buses with trolleybuses in public transport contributes to a significant reduction in the emission of nitrogen oxides, non-methane volatile organic compounds and particulate matter, while increasing the emission of sulphur dioxide, both annually and in the life cycle of these vehicles. The use of trolleybuses also contributes to a significant reduction in the damage costs due to the emission of air pollutants from public transport in urban centres, amounting to dozen thousands of Euro annually. 

However, the conducted calculations showed that, despite the lower unit emission of carbon dioxide from trolleybuses compared to diesel buses, taking into account the differences in the parameters of the operated routes (including their length generated by access to the necessary technical infrastructure), the number of trips (e.g., related to a different number of seats in vehicles) and the sum of kilometres covered on the same route, trolleybuses may be characterised by a much higher emission of this greenhouse gas (by approximately 20% per year on the tested lines). This is related to an unfavourable structure of electricity production in Poland and basing it on high-emission coal sources, with a small share of renewable energy sources. Obviously, the emission and concentration of pollutants present a heterogeneous spatial distribution, where city dwellers may be more exposed to pollution emitted by vehicles on the streets, while the emission of energy generation can occur in remote areas, increasing the chance of pollutants dispersion in the atmosphere before they reach densely populated areas.

In order for trolleybuses and electric buses to become truly green means of transport and a potential long-term solution to sustainable urban mobility, decisive actions need to be taken outside the transport sector, in particular including changes to the electricity system and transition to renewable energy sources. Further research is also needed to estimate the actual emissions of pollutants related not only to the use, but also to the production and disposal of vehicles. 

The conducted study allows us to formulate recommendations:

1. The unfavourable energy mix related to the excessive use of fossil fuels in the production of electricity does not detract from the benefits of using electric public transport.
2. Despite the increasingly higher exhaust emission standards for city buses, the benefits of operating trolleybuses with on-board batteries on the same line, in the same operating regime, are significant.
3. In the case of a city with an extensive trolleybus traction network infrastructure, it is beneficial to increase the share of trolleybus transport using on-board batteries (without the need to expand the trolleybus traction infrastructure).

## Figures and Tables

**Table 1:** Emission factors for pollutant generated by diesel buses depending on the EURO standard.

EURO Standard	Benchmark Value [g/kWh]		
	NHMC/NMVOC	NO_x_	PM
EURO 1	1.10	8.00	0.36
EURO 2	1.10	7.00	0.15
EURO 3	0.66	5.00	0.10
EURO 4	0.46	3.50	0.02
EURO 5	0.46	2.00	0.02
EURO 6	0.13	0.40	0.01

**Table 2:** Simulations of the annual emissions of pollutants from public transport and emissions in the life cycle of vehicles on the tested lines in different variants and their comparison with the emissions generated in the current operation.

Line	Variant	Annual Emission of Pollutants [Kg]					Emission of Pollutants in the Life Cycle of a Vehicle [Mg]				
		CO_2_	SO_2_	NHMC/ NMVOC	NO_x_	PM	CO_2_	SO_2_	NHMC/ NMVOC	NO_x_	PM
32	V_1_	479,157.93 *+70,230.73*	0.00 *−290.49*	822.43 *+818.66*	6258.74 *+5930.52*	33.95 *+18.86*	5749.90 *−2428.64*	0.00 *−5.81*	9.87 *+9.79*	75.10 *+68.54*	0.41 *+0.11*
170	V_2_	294,562.87 *−50,562.17*	209.25 *+209.25*	2.72 *−589.70*	236.42 *−4271.95*	10.87 *−13.59*	5891.26 *+1749.76*	4.19 *+4.19*	0.05 *−7.06*	4.73 *−49.37*	0.22 *−0.07*
181	V_3_	747,671.79 *+30,624.57*	0.00 *−274.69*	1284.25 *+713.69*	5578.88 *+3111.49*	55.24 *+30.81*	8972.06 *−2730.49*	0.00 *−5.49*	15.41 *−8.54*	111.58 *+75.76*	1.10 *+0.50*
	V_4_	694,331.76 *−22,715.46*	492.53 *+217.84*	4.60 *−565.96*	556.97 *−2221.06*	27.62 *−613.64*	13,886.64 *+2184.09*	9.85 *+4.36*	0.09 *−6.78*	11.14 *−24.68*	0.55 *−0.05*

Explanation: The differences in emissions in a given variant in relation to the emissions generated with the current operation of a given line are shown in italics.

**Table 3:** Simulations of the damage costs due to the emission of air pollutants from public transport on the analysed lines in various variants and their comparison with the costs generated in the current operation.

Line	Variant	Annual Damage Costs Due to the Emission of Air Pollutants [€]					Damage Costs Due to the Emission of Air Pollutants in the Life Cycle of Vehicles [Thous. €]				
		SO_2_	NHMC/ NMVOC	NO_x_	PM	∑	SO_2_	NHMC/ NMVOC	NO_x_	PM	∑
32	V_1_	0.00 *−2382.02*	575.70 *+573.06*	92,003.48 *+87,178.65*	546.60 *+303.65*	93,125.78 *+85,673.34*	0.00 *−47.64*	6.91 *+6.86*	1104.04 *+1007.54*	6.56 *+1.70*	1117.51 *+968.46*
170	V_2_	1715.85 *+1715.85*	1.90 *−412.79*	3475.37 *−62,797.67*	175.01 *−218.80*	5368.13 *−61,713.41*	34.32 *+34.32*	0.04 *−4.94*	69.51 *−725.77*	3.50 *−1.23*	107.37 *−697.62*
181	V_3_	0.00 *−2252.46*	898.98 *+499.59*	82,009.54 *+41,172.50*	889.36 *+248.10*	83,797.88 *+39,667.73*	0.00 *−45.05*	10.79 *+5.98*	984.11 *+457.53*	10.67 *+0.99*	1005.57 *+419.45*
	V_4_	4038.75 *+1786.29*	3.22 *−396.17*	8187.46 *−32,649.58*	444.68 *−246.58*	12,674.11 *−31,456.04*	48.47 *+3.42*	0.06 *−4.75*	163.75 *−362.83*	8.89 *−0.76*	221.17 *−364.95*

Explanation: The differences in costs in a given variant in relation to the costs generated in the current operation of the line are indicated in italics.

**Table 4:** Characteristics of the functioning of the analysed lines 32 (170) and 181 (source: Own elaboration based on unpublished exploitation materials).

Line		Length of the Route in One Direction [Km]	Max. of the Length of the Route Under the Overhead Line in One Direction [Km]	The Number of Trips in Both Directions during One Week	The Total Number of Vehicle Kilometres during One Week [Km]	Max. Number of Vehicles Used on the Line	Vehicle Curb Weight (Total Weight) [Kg]	Engine Power	Energy Consumption Per 100 Km [Kwh or Liter of Diesel Fuel]	Total Number of Seats
32	trolleybus	9.9–10.4 ^1^	4.3	782 ^2^	7255	7	13,550 (18,745)	170 kW	143 kWh	76
170	bus	7.6–8.4 ^3^	-	773 ^2^	5226	6	10,800 (18,000)	180 kW	47.39 l l	105 ^4^
181	bus	12.8	-	425 ^5^	3915	3	16,795 (28,000)	220 kW	60.6 l l	138 ^6^
	trolleybus		6.1 + 2.2 ^7^	230 ^5^ 138 ^8^	4937	6	13,185 (18,745) 19,125 (28,000)	175 kW (12 m) ^9^ 250 kW (18 m) ^9^	199 kWh	78 121

^1^ the difference in distance in one direction is 0.5 km. ^2^ about half of the trips are shortened to the Pogórze Dolne loop, rest of the trips finish at Pogórze Dolne Złota loop, a few selected trips on the shortened route or trips from the depot and to the depot. ^3^ the difference in distance in one direction is 0.8 km. ^4^ line 170 was usually served by Solaris Urbino 12 buses manufactured in 2005–2015 with the Euro 3–5 exhaust emission standard. ^5^ the number of trips in one direction on weekdays. ^6^ articulated buses used on line 181 meet the Euro 5 exhaust emission standard. ^7^ the middle section of the route, 4.7 km long, has no overhead contact line. ^8^ the number of trips in one direction at weekends served by 12-m long trolleybuses. ^9^ on Saturdays and Sundays, the line is served only by trolleybuses with a length of 12 m; on other days there are buses and trolleybuses with a length of 18 m.

**Table 5:** Unit emission of pollutants from means of public transport on the analysed lines.

Line	Vehicle Type	Unit Emission of Pollutants [g/km]				
		CO_2_	SO_2_	NHMC/NMVOC	NO_x_	PM
32	trolley bus	1083.94	0.77	0.01	0.87	0.04
170	bus	1270.10	-	2.18	16.59	0.09
181	trolley bus	1508.42	1.07	0.01	1.21	0.06
	bus	1624.30	-	2.79	12.12	0.12

**Table 6:** Annual emission of pollutants and emission in the life cycle of public transport vehicles on the analysed lines.

Line	Vehicle Type	Annual Emission of Pollutants [Kg]					Emission of Pollutants in the Life Cycle of a Vehicle [Mg]				
		CO_2_	SO_2_	NHMC/ NMVOC	NO_x_	PM	CO_2_	SO_2_	NHMC/ NMVOC	NO_x_	PM
32	trolleybus	408,927.20	290.49	3.77	328.22	15.09	8178.54	5.81	0.08	6.56	0.30
170	bus	345,125.04	0.00	592.42	4508.37	24.46	4141.50	0.00	7.11	54.10	0.29
181	trolleybus	387,247.62	274.69	2.57	310.64	15.40	7744.95	5.49	0.05	6.21	0.31
	bus	329,799.60	0.00	567.99	2467.39	24.43	3957.60	0.00	6.82	29.61	0.29

**Table 7:** Damage costs of the emission of air pollutant from public transport on the analysed lines.

Line	Vehicle Type	Annual Damage Costs of the Emission of Air Pollutants [€]					Damage Costs of the Emission of Air Pollutants in the Life Cycle of Vehicles [Thous. €]				
		SO_2_	NHMC/ NMVOC	NO_x_	PM	∑	SO_2_	NHMC/ NMVOC	NO_x_	PM	∑
32	trolleybus	2382.02	2.64	4824.83	242.95	7452.44	47.64	0.05	96.50	4.86	149.05
170	bus	0.00	414.69	66,273.04	393.81	67,081.54	0.00	4.98	795.28	4.73	804.99
181	trolleybus	2252.46	1.80	4566.41	247.94	7068.61	45.05	0.04	91.33	4.96	141.38
	bus	0.00	397.59	36,270.63	393.32	37,061.54	0.00	4.77	435.25	4.72	444.74

## Data Availability

Not applicable.
